# Risk factors for COVID-19 hospitalization and mortality in patients with chronic kidney disease: a nationwide cohort study

**DOI:** 10.1093/ckj/sfad283

**Published:** 2023-11-28

**Authors:** Angelica Artborg, Aurora Caldinelli, Julia Wijkström, Alexandra Nowak, Michael Fored, Maria Stendahl, Marie Evans, Helena Rydell

**Affiliations:** Department of Clinical Science, Intervention and Technology (CLINTEC), Karolinska Institutet, Stockholm, Sweden; Department of Renal Medicine, Karolinska University Hospital, Stockholm, Sweden; Department of Clinical Science, Intervention and Technology (CLINTEC), Karolinska Institutet, Stockholm, Sweden; University of Milano-Bicocca, Department of Statistics and Quantative Methods, Milan, Italy; Department of Clinical Science, Intervention and Technology (CLINTEC), Karolinska Institutet, Stockholm, Sweden; Department of Renal Medicine, Karolinska University Hospital, Stockholm, Sweden; Department of Clinical Science, Intervention and Technology (CLINTEC), Karolinska Institutet, Stockholm, Sweden; Department of Renal Medicine, Karolinska University Hospital, Stockholm, Sweden; Department of Medicine, Karolinska Institutet, Stockholm, Sweden; Department of Internal Medicine, Ryhov Hospital, Jönköping, Sweden; Swedish Renal Register, Jönköping, Sweden; Department of Clinical Science, Intervention and Technology (CLINTEC), Karolinska Institutet, Stockholm, Sweden; Department of Renal Medicine, Karolinska University Hospital, Stockholm, Sweden; Swedish Renal Register, Jönköping, Sweden; Department of Clinical Science, Intervention and Technology (CLINTEC), Karolinska Institutet, Stockholm, Sweden; Department of Renal Medicine, Karolinska University Hospital, Stockholm, Sweden; Swedish Renal Register, Jönköping, Sweden

**Keywords:** CKD, COVID-19, dialysis, mortality, risk factors

## Abstract

**Background:**

Several studies have demonstrated an increased risk of severe coronavirus disease 2019 (COVID-19) in chronic kidney disease (CKD) patients. However, few have investigated the impact of CKD stage and dialysis modality. The primary aim of this study was to investigate the association between CKD stage, dialysis modality and risk of severe COVID-19. Secondly, we aimed to study the impact of comorbidities and drugs on the risk of severe COVID-19 in the CKD population.

**Methods:**

This nationwide observational study was based on data from the Swedish Renal Registry and three other national registries. Patients with non-dialysis CKD stage 3b–5 or dialysis on 1 January 2020 were included and followed until 31 December 2021. The primary outcome was COVID-19 hospitalization; the secondary outcome was COVID-19 mortality. Associations were investigated using logistic regression models, adjusting for confounders.

**Results:**

The study population comprised 7856 non-dialysis CKD patients and 4018 dialysis patients. The adjusted odds ratios (aOR) for COVID-19 hospitalization and mortality were highest in the dialysis group [aOR 2.24, 95% confidence interval (CI) 1.79–2.81; aOR 3.10, Cl 95% 2.03–4.74], followed by CKD 4 (aOR 1.33, 95% CI 1.05–1.68; aOR 1.66, Cl 95% 1.07–2.57), as compared with CKD 3b. No difference in COVID-19 outcomes was observed between patients on hemodialysis and peritoneal dialysis. Overall comorbidity burden was one of the strongest risk factors for severe COVID-19 and the risk was also increased in patients prescribed insulin, proton pump inhibitors, diuretics, antiplatelets or immunosuppressants.

**Conclusions:**

Worsening CKD stage and comorbidity are independent risk factors for severe COVID-19 in the Swedish CKD population.

KEY LEARNING POINTS **What was known:**

- Chronic kidney disease (CKD) is a key risk factor for severe coronavirus disease 2019 (COVID-19). Few studies have investigated the risk of severe COVID-19 associated with different CKD stages, type of dialysis modality and comorbidity in the CKD population.

**This study adds:**

- In our nationwide cohort of CKD patients with estimated glomerular filtration rate <45 mL/min we showed that worsening CKD stage and dialysis are independent risk factors for COVID-19-related hospitalization and mortality.
- Overall comorbidity burden was one of the most prominent risk factors for severe COVID-19 in this population.

**Potential impact:**

- Awareness of risk factors for severe COVID-19 in the CKD population could reduce mortality by guiding healthcare recourses such as vaccinations and novel treatments to patients at particular risk.

## INTRODUCTION

Coronavirus disease 2019 (COVID-19) emerged in China in December 2019, leading to a public health crisis as the infection spread globally [1]. With surges of new virus variants occurring continuously, COVID-19 still puts a significant strain on healthcare systems. Over the course of the pandemic, Sweden has not been spared. The first COVID-19 case in Sweden was detected on 31 January 2020, and community transmission was observed in the beginning of March 2020 [2, 3]. By the end of December 2022, 2.68 million COVID-19 cases and 22 427 COVID-19-related deaths had been officially recorded in Sweden, corresponding to 2.2 deaths per 1000 inhabitants [4]. In contrast to many other countries, Sweden did not employ a strict lockdown policy as the pandemic advanced. Instead, the national preventive strategy was based on voluntary measures such as social distancing and self-isolation in case of potential COVID-19 symptoms. COVID-19 vaccinations were launched in December 2021, with certain risk groups being prioritized in a stepwise manner [5]. Vaccinations of dialysis patients were initiated in March to April 2021 and patients with chronic kidney disease (CKD) were included in April [6, 7].

Since the emergence of the pandemic, CKD has been identified as one of the major risk factors for COVID-19 severity [8, 9]. However, most reports of COVID-19 in the CKD population have been small in scale or primarily focused on end-stage CKD, while comparisons with earlier CKD stages are scarce [8, 10, 11]. Moreover, as advanced age and multimorbidity are common among CKD patients, the specific influence of CKD beyond the burden of associated comorbidities on COVID-19 prognosis remains unclear.

The primary aim of this study was to determine the association between CKD stage, as well as dialysis modality, and risk of COVID-19-related hospitalization and mortality in a large cohort of patients with CKD stage 3b–5 and dialysis. The secondary aim was to determine the association between selected risk factors and severe COVID-19 in this population.

## MATERIALS AND METHODS

### Study population

**Figure 1:**
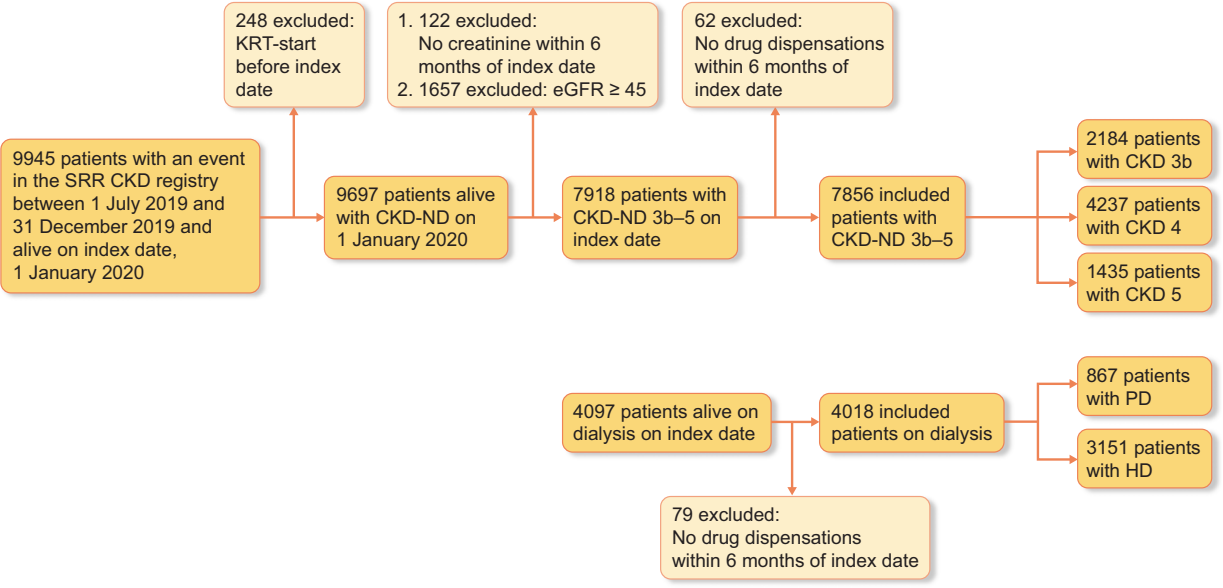
Inclusion and exclusion of patients with CKD stage 3b–5 or dialysis.

The study was carried out as a nationwide observational study based on data from the Swedish Renal Registry (SRR), a registry for nephrology-referred patients in Sweden. The database has a national coverage; >97% of dialysis patients are registered and about 80% of patients initiating dialysis are followed up during nephrology outpatient care [12, 13]. Adult patients (≥18 years) registered in SRR with non-dialysis dependent CKD (CKD-ND) stage 3b–5 or ongoing dialysis on 1 January 2020 (index date) were included in the analysis. Patients were excluded if no drugs had been dispensed within 6 months of index date (Fig. 1). In the CKD-ND population, we also excluded patients with no registered serum creatinine or prior kidney replacement therapy (KRT) within 6 months of index date. Patients were followed until either death, 31 December 2021 or having fulfilled study outcome (whichever occurred first). Only a small number of patients moved abroad during the study (*n* = 23) and there was no loss to follow-up among those that remained in Sweden. The study was approved by the regional ethics committee in Stockholm (EPN Stockholm 2018/1591-31/2, 2020-04778 and 2021-0067).

### Data collection

Data from the SRR were individually linked to the National Patient Register (NPR), the Prescribed Drug Register (PDR) and the Cause of Death Register (CDR) using a personal identification number and were then anonymized. CKD specific characteristics were recorded from the SRR. Primary and secondary diagnoses from hospital admissions and specialist outpatient visits coded according to the International Classification of Diseases (ICD)-10 were collected from the NPR. Information on drug dispensations from pharmacies were collected from the PDR. All causes of deaths, including dates, were collected from the CDR.

### Outcomes

The primary outcome was hospital admission due to COVID-19, or death due to COVID-19 before hospital admission. Overall mortality due to COVID-19 was the secondary outcome. COVID-19-related death was defined as a laboratory-verified diagnosis of COVID-19 (ICD-10 code U071) as the main or contributing cause of death in the CDR, whereas for the definition of COVID-19-related hospitalization we only included the first hospital admission with a diagnosis of COVID-19 recorded in either the first or second position in the NPR.

### CKD exposure and risk factors

Our primary objective was to study the association between CKD stage/dialysis and severe COVID-19. The last creatinine value registered in SRR before index date was used to calculate the estimated glomerular filtration rate (eGFR) according to the CKD Epidemiology Collaboration equation [14], and patients were divided in CKD stages 3b (eGFR 44–30 mL/min/1.73 m^2^), 4 (eGFR 29–15 mL/min/1.73 m^2^) and 5 (GFR <15 mL/min/1.73 m^2^). Dialysis patients were divided into groups based on dialysis modality at index date—peritoneal dialysis (PD) or hemodialysis (HD). Patients were presumed to remain in their appointed groups throughout the observational period. Information on dialysis therapy initiation, changes of KRT modality and creatinine value at last outpatient nephrologist visit was also attained from the SRR.

The other risk factors of interest were age, sex, primary kidney disease, separate comorbid diseases, drug exposure and Charlson comorbidity index (CCI) score. Information on age, sex and primary kidney disease (as diagnosed by the treating nephrologist) was extracted from SRR. The comorbidities comprising the CCI were recorded from the NPR using all available ICD-10 codes up to index date (Supplementary data, Table S1) [15, 16]. As our population consisted exclusively of CKD patients, we did not include kidney disease in the CCI score. Drug exposure was defined as at least one dispensation within 6 months of index date registered in the PDR. The Anatomical Therapeutic Chemical (ATC) codes used for the classification of drugs are listed in Supplementary data, Table S2. There were no missing data on any of the included variables.

### Statistical analysis

Categorial variables were reported as numbers and percentages, while continuous variables were reported as median and interquartile range (IQR). The incidence of COVID-19-related hospitalization (or death due to COVID-19 before hospitalization) and mortality in all subgroups were estimated using the cumulative incidence function, with death of other causes as a competing event.

To study the association between each risk factor and the risk of COVID-19-related outcomes, univariate and multivariate binary logistic regression models were applied with adjustments for different confounding factors in different analyses based on *a priori* assumptions on their respective relationships. Age, sex, primary kidney disease, CCI score, CKD stage and/or dialysis vintage were included as possible confounding factors, as appropriate in each of the analyses. The results from the logistic regression analyses were presented as crude odds ratios (OR) and adjusted odds ratios (aOR), with 95% confidence intervals (CIs) and *P*-values. Statistical significance was defined as *P* ≤ .05. All data were analysed with SAS (version 9.4).

## RESULTS

A total of 11 874 patients were included, 7856 patients with CKD-ND stage 3b–5 and 4018 patients on dialysis. In the CKD-ND population 28% (*N* = 2184) were classified as CKD stage 3b, 54% (*N* = 4237) as stage 4 and 18% (*N* = 1435) as stage 5. Dialysis patients were either on HD (78%, *N* = 3151) or PD (22%, *N* = 867) (Fig. 1).

Patients’ characteristics, including comorbidities and medications, are shown in Table 1. In the baseline population 64% (*N* = 7543) were men, and the median age was 73 years (IQR 63–79 years). CKD-ND patients were in general older than dialysis patients. Hypertension and renovascular disease were the main causes of kidney disease in CKD-ND patients (29.8%, 31.6% and 28.2% in CKD stage 3b, 4 and 5, respectively), whereas diabetic kidney disease was the most common cause in dialysis patients (25%). The proportion of patients with diabetic kidney disease increased with increasing CKD stage. A CCI score ≥5 was more common in dialysis patients (24.2%) than in CKD-ND patients (20.7%, 20.1% and 17.8% in CKD stage 3b, 4 and 5, respectively). Accordingly, dialysis patients had a higher incidence of cardiovascular disease, diabetes and pulmonary disease, than patients in earlier CKD stages.

**Table 1:** Baseline characteristics of patients with CKD stage 3b–5 or dialysis.

	Total cohort	CKD 3b	CKD 4	CKD 5	Dialysis, total	HD	PD
Number of patients	11 874	2184	4237	1435	4018	3151	867
Age, years, median (IQR)	73 (63–79)	72 (63–78)	75 (66–81)	74 (65–81)	70 (58–77)	70 (58–77)	70 (57–77)
Sex, % (*N*)							
Male	63.5 (7543)	63.3 (1383)	62.2 (2636)	61.3 (879)	65.8 (2645)	64.3 (2027)	71.3 (618)
Primary kidney disease, % (*N*)							
Glomerulonephritis	12.1 (1440)	12.5 (274)	9.3 (392)	9.7 (139)	15.8 (635)	15.0 (473)	18.7 (162)
Diabetic kidney disease	20.3 (2406)	14.9 (325)	18.8 (795)	20.6 (295)	24.7 (991)	25.3 (797)	22.4 (194)
Hypertensive nephrosclerosis or renovascular disease	26.7 (3169)	29.8 (650)	31.6 (1339)	28.2 (404)	19.3 (776)	18.5 (583)	22.3 (193)
Adult polycystic kidney disease	6.0 (708)	6.0 (132)	4.4 (187)	6.8 (98)	7.2 (291)	7.3 (230)	7.0 (61)
Pyelonephritis	4.0 (471)	3.1 (67)	3.9 (167)	5.2 (75)	4.0 (162)	3.9 (123)	4.5 (39)
Other specified kidney disease	19.6 (2331)	20.4 (445)	19.5 (828)	18.8 (270)	19.6 (788)	20.7 (652)	15.7 (136)
Unknown kidney disease	11.3 (1348)	13.3 (291)	12.5 (528)	10.7 (154)	9.3 (375)	9.3 (293)	9.5 (82)
Dialysis vintage, median (IQR)							
Years since start of dialysis						2 (1–4)	1 (0–2)
Comorbidities, % (*N*)							
Coronary artery disease	28.2 (3353)	25.5 (556)	27.9 (1183)	24.8 (356)	31.3 (1258)	31.6 (996)	30.2 (262)
Congestive heart failure	28.1 (3339)	23.5 (513)	26.9 (1138)	22.9 (329)	33.8 (1359)	35.7 (1124)	27.1 (235)
Peripheral vascular disease	9.8 (1169)	7.3 (159)	6.4 (270)	6.1 (87)	16.3 (653)	18.6 (586)	7.7 (67)
Cerebrovascular disease	17.7 (2098)	16.6 (362)	17.0 (722)	15.9 (228)	19.6 (786)	20.2 (637)	17.2 (149)
Hemiplegia	1.7 (203)	1.5 (32)	1.4 (60)	1.4 (20)	2.3 (91)	2.4 (75)	1.8 (16)
Dementia	1.2 (144)	0.9 (19)	1.3 (53)	1.5 (21)	1.3 (51)	1.4 (44)	0.8 (7)
Chronic pulmonary disease	12.7 (1504)	12.3 (269)	11.9 (504)	10.0 (143)	14.6 (588)	15.3 (483)	12.1 (105)
Diabetes without complications	39.2 (4658)	37.0 (809)	38.9 (1647)	36.2 (520)	41.9 (1682)	43.1 (1359)	37.3 (323)
Diabetes with end-organ damage	34.0 (4038)	30.4 (663)	32.3 (1370)	32.8 (470)	38.2 (1535)	39.3 (1247)	33.2 (288)
Connective tissue disorder	10.3 (1219	12.7 (278)	9.8 (417)	8.4 (121)	10.0 (403)	10.9 (342)	7.0 (61)
Mild liver disease	2.6 (327)	2.2 (48)	1.9 (82)	1.8 (26)	4.3 (171)	4.5 (143)	3.2 (28)
Moderate to severe liver disease	1.8 (209)	2.0 (43)	1.5 (63)	1.0 (14)	2.2 (89)	2.4 (75)	1.6 (14)
Peptic ulcer disease	7.4 (879)	6.2 (135)	6.5 (276)	5.8 (83)	9.6 (385)	10.3 (326)	6.8 (59)
Leukemia, lymphoma or multiple myeloma	3.3 (388)	3.0 (66)	2.9 (124)	3.0 (43)	3.9 (155)	3.8 (120)	4.0 (35)
Solid tumor without metastasis	29.6 (3514)	30.9 (674)	30.2 (1278)	29.3 (420)	28.4 (1142)	28.9 (912)	26.5 (230)
Solid metastatic tumor	3.0 (350)	3.1 (68)	3.1 (130)	3.1 (44)	2.7 (108)	2.9 (90)	2.1 (18)
HIV	0.2 (28)	0.2 (4)	0.1 (3)	0.3 (4)	0.4 (17)	0.4 (13)	0.5 (4)
CCI score							
Null	16.6 (1976)	19.4 (423)	17.5 (740)	21.7 (311)	12.5 (502)	10.0 (314)	21.7 (188)
1–2	30.8 (3659)	31.7 (692)	32.4 (1375)	30.2 (433)	28.8 (1159)	29.1 (918)	27.8 (241)
3–4	30.9 (3663)	28.2 (617)	29.9 (1267)	30.4 (436)	33.4 (1343)	34.0 (1971)	31.4 (272)
≥5	21.7 (2576)	20.7 (452)	20.1 (855)	17.8 (255)	24.2 (1014)	26.9 (848)	19.1 (166)
Medication use, % (*N*)							
Systemic corticosteroid	21.4 (2536)	28.9 (632)	20.3 (862)	15.4 (221)	20.4 (221)	21.5 (679)	16.4 (142)
Other immunosuppressant drugs	11.5 (1370)	21.5 (469)	9.1 (384)	5.4 (78)	10.9 (439)	11.4 (358)	9.3 (81)
ACE-I or ARB	56.8 (6741)	67.9 (1482)	66.0 (2795)	51.2 (735)	43.0 (1729)	41.8 (1316)	47.6 (413)
Beta blocker	68.1 (8091)	63.1 (1379)	67.0 (2839)	70.4 (1010)	71.3 (2863)	70.4 (2219)	74.3 (644)
Calcium channel blocker	58.4 (6936)	53.5 (1169)	60.8 (2576)	71.6 (1027)	53.9 (2164)	51.7 (1629)	61.7 (535)
Alfa receptor blocker	17.1 (2035)	12.7 (278)	15.2 (645)	21.3 (305)	20.1 (807)	18.8 (592)	24.8 (215)
Diuretic	62.1 (7373)	53.7 (1173)	62.9 (2665)	70.6 (1013)	62.8 (2522)	57.3 (1807)	82.5 (715)
Antiplatelet agent	36.0 (4277)	31.8 (695)	33.9 (1436)	35.1 (503)	40.9 (1643)	42.1 (1327)	36.4 (316)
Warfarin or DOAC	18.1 (2153)	23.4 (510)	21.3 (903)	15.2 (218)	13.0 (522)	12.9 (408)	13.1 (114)
Oral antidiabetic drug	14.3 (1699)	19.1 (418)	18.5 (782)	13.6 (195)	7.6 (304)	7.4 (233)	8.2 (71)
Insulin	26.5 (3144)	25.9 (565)	28.3 (1197)	24.2 (347)	25.8 (1035)	25.7 (811)	25.8 (224)
Antidepressant drug	16.2 (1926)	13.8 (301)	14.6 (618)	14.0 (201)	20.1 (806)	21.2 (669)	15.8 (137)
Statin	59.6 (7073)	65.4 (1429)	63.5 (2689)	59.5 (854)	52.3 (2101)	50.7 (1596)	58.2 (505)
Proton-pump inhibitor	43.6 (5173)	36.0 (787)	37.6 (1591)	35.6 (511)	56.8 (2284)	58.6 (1845)	50.6 (439)

ARB, angiotensin II-receptor blocker; DOAC, direct oral anticoagulants.

### CKD stage and dialysis modality

**Figure 2:**
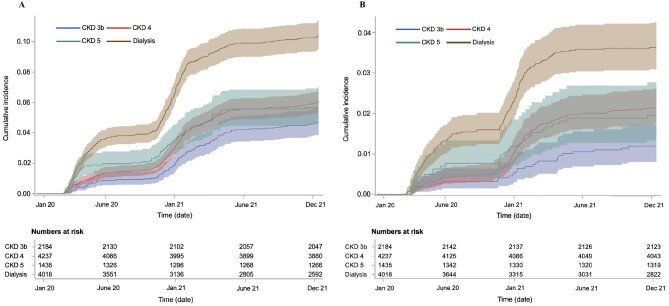
Unadjusted cumulative incidence with 95% CIs of a first COVID-19 hospitalization or COVID-19 death before hospitalization (**A**) and COVID-19 mortality (**B**) in adult patients with CKD stage 3b (blue line), stage 4 (red line), stage 5 (green line) and dialysis (brown line) during January 2020 to December 2021.

Patients were followed for a median of 654 days for COVID-19-related hospitalization and 673 days for COVID-19-related death (Supplementary data, Table S3). The cumulative incidences of a first COVID-19-associated hospitalization (including death due to COVID-19 before hospitalization) and COVID-19-related mortality are presented in Fig. 2A and B, respectively. In total, 1.2% (*N* = 26), 2.2% (*N* = 91), 2.0% (*N* = 28) and 3.6% (*N* = 146) of the patients with CKD stage 3b, 4, 5 and dialysis died from COVID-19 during the first 2 years of the pandemic (Table 2). COVID-19-related mortality accounted for 7%–12% of overall mortality these years, with the proportion being highest in dialysis patients (Supplementary data, Table S4). In adjusted analyses, dialysis patients had the highest risk of COVID-19-related hospitalization compared with CKD stage 3b (aOR 2.24, 95% CI 1.79–2.81), followed by CKD stage 4 (aOR 1.33, 95% CI 1.05–1.68) and CKD stage 5 (aOR 1.27, 95% CI 0.94–1.71) (Table 2). Likewise, the highest risk for COVID-19-related mortality was observed for dialysis patients (aOR 3.10, 95% CI 2.03–4.74), followed by patients with CKD stage 4 (aOR 1.66, Cl 95% 1.07–2.57) and CKD stage 5 (aOR 1.59, 95% CI 0.92–2.7).

**Table 2:** COVID-19 outcomes and associations with CKD stage and dialysis modality in patients with CKD stage 3b–5 or dialysis.

	Number of events, % (*N*)		Incidence rate per 10 000 person-years (95% CI)		Univariate				Multivariate			
	Hospitalization or death before hospitalization	Death	Hospitalization or death before hospitalization	Death	Hospitalization or death before hospitalization		Death		Hospitalization or death before hospitalization		Death	
Characteristic					OR (95% CI)	*P*-value	OR (95% CI)	*P*-value	Adjusted OR (95% CI)	*P*-value	Adjusted OR (95% CI)	*P*-value
CKD stage^a^												
3b	4.7 (102)	1.2 (26)	0.66 (0.55–0.81)	0.17 (0.12–0.25)	Ref.		Ref.		Ref.		Ref.	
4	6.1 (257)	2.2 (91)	0.88 (0.78–0.99)	0.31 (0.25–0.38)	1.33 (1.05–1.68)	.02	1.82 (1.17–2.83)	<.001	1.33 (1.05–1.68)	.02	1.66 (1.07–2.57)	.02
5	5.7 (82)	2.0 (28)	0.86 (0.69–1.06)	0.29 (0.20–0.42)	1.24 (0.92–1.67)	.16	1.65 (0.96–2.83)	.07	1.27 (0.94–1.71)	.12	1.59 (0.92–2.72)	.10
Dialysis	10.4 (419)	3.6 (146)	1.79 (1.62–1.97)	0.62 (0.53–0.73)	2.35 (1.88–2.93)	<.001	3.13 (2.05–4.77)	<.001	2.24 (1.79–2.81)	<.001	3.10 (2.03–4.74)	<.001
Dialysis modality^b^												
PD	8.2 (71)	3.7 (32)	1.81 (1.63–2.02)	0.59 (0.49–0.71)	Ref.		Ref.		Ref.		Ref	
HD	11.0 (348)	3.6 (114)	1.34 (1.06–1.69)	0.60 (0.43–0.85)	1.39 (1.07–1.82)	.02	0.98 (0.66–1.46)	.92	1.30 (0.99–1.71)	.06	0.84 (0.56–1.28)	.42

Clinical outcomes are due to COVID-19. *P* is considered significant at *P* ≤ .05.

^a^Multivariate analysis adjusted for age, sex, primary kidney disease and CCI score.

^b^Multivariate analysis adjusted for age, sex, primary kidney disease, CCI score and dialysis vintage.

When restricting our analyses to dialysis patients, HD patients had significantly higher unadjusted ORs for COVID-19-associated hospitalization compared with PD patients (OR 1.39, 95% CI 1.07–1.82) (Table 2). After adjustment for confounding factors the magnitude of the association dropped slightly and was no longer statistically significant (aOR 1.30, 95% CI 0.99–1.71). There was no difference in COVID-19-related mortality between HD and PD patients (aOR 0.84, 95% CI 0.56–1.28).

### Comorbidities and primary kidney disease

Associations between comorbidity, primary kidney disease, and severe COVID-19 are outlined in Table 3. A higher CCI score was associated with both COVID-19-related hospitalization and death. For patients with a CCI score ≥5 the aORs were 2.22 (95% CI 1.70–2.90) for hospitalization and 6.47 (95% CI 3.22–12.98) for mortality compared with CCI score 0. Most of the individual comorbidities were associated with a higher risk of severe COVID-19. The comorbidities with the highest risk of COVID-19-related hospitalization were diabetes with end-organ damage (aOR 1.59, 95% CI 1.38–1.83) and severe liver disease (aOR 1.59, 95% CI 1.03–2.45). Among the comorbidities most strongly associated with mortality were dementia (aOR 2.44, 95% CI 1.26–4.71) and coronary artery disease (aOR 2.02, 95% CI 1.59–2.57). Type of primary kidney disease was not significantly associated with any of the two outcomes in the multivariate analysis.

**Table 3:** Univariate and multivariate associations between comorbidities, primary kidney diseases and COVID-19 outcomes in patients with CKD stage 3b–5 or dialysis.

	Univariate				Multivariate			
	Hospitalization or death before hospitalization		Death		Hospitalization or death before hospitalization		Death	
Characteristic	OR (95% CI)	*P*-value	OR (95% CI)	*P*-value	Adjusted OR (95% CI)	*P*-value	Adjusted OR (95% CI)	*P*-value
Age and sex^a^								
Age	1.00 (0.99–1.00)	.15	1.04 (1.03–1.05)	<.001	0.92 (0.79–1.07)	.28	1.04 (1.03–1.05)	<.001
Women	0.88 (0.76–1.02)	.10	0.81 (0.63–1.05)	.11	0.90 (0.78–1.04)	.17	0.86 (0.67–1.10)	.23
CCI score^a^								
Null	Ref.		Ref.		Ref.		Ref.	
1–2	1.43 (1.12–1.83)	.004	4.57 (2.26–9.14)	<.001	1.47 (1.14–1.89)	.003	3.56 (1.77–7.15)	<.001
3–4	1.68 (1.32–2.13)	<.001	5.82 (2.93–11.54)	<.001	1.73 (1.34–2.25)	<.001	3.92 (1.95–7.87)	<.001
≥5	2.13 (1.66–2.72)	<.001	9.93 (5.02–19.63)	<.001	2.22 (1.70–2.90)	<.001	6.47 (3.22–12.98)	<.001
Comorbidities^b^								
Coronary artery disease	1.47 (1.27–1.70)	<.001	2.56 (2.02–3.23)	<.001	1.44 (1.24–1.68)	<.001	2.02 (1.59–2.57)	<.001
Congestive heart failure	1.45 (1.26–1.68)	<.001	2.05 (1.62–2.59)	<.001	1.38 (1.19–1.61)	<.001	1.59 (1.25–2.02)	<.001
Peripheral vascular disease	1.29 (1.04–1.59)	.02	1.84 (1.35–2.53)	<.001	1.10 (0.88–1.37)	.40	1.49 (1.08–2.06)	.02
Cerebrovascular disease	1.18 (0.99–1.40)	.06	1.93 (1.49–2.50)	<.001	1.15 (0.97–1.38)	.12	1.60 (1.23–2.08)	<.001
Hemiplegia	1.02 (0.60–1.74)	.94	1.65 (0.82–3.38)	.17	0.93 (0.55–1.59)	.80	1.62 (0.79–3.35)	.19
Dementia	1.39 (0.80–2.42)	.25	3.04 (1.58–5.84)	<.001	1.37 (0.79–2.41)	.27	2.44 (1.26–4.71)	.008
Chronic pulmonary disease	1.39 (1.15–1.68)	<.001	1.75 (1.30–2.34)	<.001	1.36 (1.12–1.64)	.002	1.59 (1.18–2.13)	.002
Diabetes without complications	1.53 (1.33–1.75)	<.001	1.72 (1.36–2.17)	<.001	1.48 (1.28–1.73)	<.001	1.64 (1.30–2.08)	<.001
Diabetes with end-organ damage	1.68 (1.46–1.93)	<.001	1.70 (1.34–2.15)	<.001	1.59 (1.38–1.83)	<.001	1.66 (1.32–2.10)	<.001
Connective tissue disorder	0.88 (0.69–1.11)	.28	1.12 (0.78–1.62)	.54	0.91 (0.71–1.15)	.42	1.13 (0.78–1.63)	.54
Mild liver disease	1.77 (1.26–2.49)	.001	1.27 (0.67–2.40)	.47	1.50 (1.06–2.12)	.02	1.49 (0.78–2.86)	.23
Moderate or severe liver disease	1.68 (1.09–2.59)	.02	1.60 (0.78–3.28)	.20	1.59 (1.03–2.45)	.04	1.73 (0.84–3.57)	.14
Peptic ulcer disease	1.35 (1.07–1.72)	.01	1.74 (1.21–2.49)	.01	1.25 (0.98–1.59)	.07	1.48 (1.03–2.12)	.04
Leukaemia, myeloma, lymphoma	0.73 (0.47–1.13)	.16	0.73 (0.34–1.55)	.41	0.69 (0.44–1.08)	.10	0.69 (0.32–1.48)	.34
Solid tumor without metastases	0.88 (0.75–1.03)	.11	1.23 (0.97–1.58)	.10	0.90 (0.77–1.06)	.20	0.95 (0.74–1.23)	.71
Solid metastatic tumor	0.65 (0.40–1.06)	.08	0.45 (0.17–1.22)	.12	0.67 (0.41–1.09)	.11	0.43 (0.16–1.16)	.10
Primary kidney disease^c^								
Glomerulonephritis	Ref.		Ref.		Ref.		Ref.	
Diabetic kidney disease	1.42 (1.11–1.82)	.005	1.92 (1.23–3.00)	.004	1.21 (0.94–1.57)	.14	1.35 (0.96–1.90)	.08
Hypertensive/renovascular kidney disease	1.04 (0.81–1.33)	.77	1.39 (0.89–2.18)	.15	1.08 (0.84–1.39)	.56	1.16 (0.81–1.67)	.43
Adult polycystic kidney disease	0.94 (0.65–1.35)	.74	1.02 (0.52–1.99)	.96	1.00 (0.69–1.44)	.99	1.04 (0.64–1.69)	.87
Pyelonephritis	0.71 (0.45–1.13	.15	0.82 (0.35–1.90)	.65	0.74 (0.47–1.19)	.22	1.03 (0.56–1.87)	.93
Other specified kidney disease	1.01 (0.78–1.32)	.96	1.29 (0.80–2.07)	.29	1.00 (0.77–1.32)	.99	0.92 (0.64–1.34)	.67
Unknown kidney disease	1.01 (0.76–1.36)	.93	1.24 (0.73–2.10)	.43	1.04 (0.77–1.40)	.81	1.15 (0.74–1.76)	.54

Clinical outcomes are due to COVID-19. *P* is considered significant at *P* ≤ .05.

^a^Multivariate analysis adjusted for age, sex, primary kidney disease and CCI score.

^b^Multivariate analysis adjusted for age, sex and CKD stage/dialysis.

^c^Multivariate analysis adjusted for age, sex and CCI score.

### Medications

In our CKD population, a higher risk of hospitalization due to COVID-19 was observed in patients prescribed insulin (aOR 1.36, 95% CI 1.12–1.64), proton pump inhibitors (PPI) (aOR 1.32, 95% CI 1.13–1.51), diuretics (aOR 1.29, 95% CI 1.01–1.37), corticosteroids (aOR 1.32, 95% CI 1.11–1.56) or other immunosuppressants (aOR 1.49, 95% CI 1.20–1.85) (Table 4). Treatment with insulin, antiplatelets and PPI were associated with increased risk of COVID-19-related mortality (aOR 1.41, 95% CI 1.04–1.90; aOR 1.29, 95% CI 1.01–1.64; and aOR 1.47, 95% CI 1.15–1.88).

**Table 4:** Univariate and multivariate associations between dispensed medications and COVID-19 outcomes in patients with CKD stage 3b–5 or dialysis.

	Univariate				Multivariate			
Characteristic	Hospitalization or death before hospitalization		Death		Hospitalization or death before hospitalization		Death	
Medications^a^	OR (95% CI)	*P*-value	OR (95% CI)	*P*-value	Adjusted OR (95% CI)	*P*-value	Adjusted OR (95% CI)	*P*-value
Systemic corticosteroid	1.27 (1.09–1.50)	.003	1.02 (0.77–1.35)	.90	1.32 (1.11–1.56)	.001	1.17 (0.87–1.56)	.31
Other immunosuppressant drugs	1.35 (1.12–1.64)	.003	0.68 (0.45–1.04)	.08	1.49 (1.20–1.85)	<.001	1.06 (0.68–1.66)	.80
ACE-I or ARB	0.89 (0.77–1.02)	.10	0.78 (0.62–0.99)	.04	0.98 (0.85–1.14)	.81	0.98 (0.77–1.25)	.87
Beta blocker	1.13 (0.97–1.31)	.13	1.18 (0.91–1.52)	.22	1.00 (0.86–1.16)	.96	0.97 (0.75–1.26)	.81
Calcium channel blocker	0.95 (0.83–1.10)	.50	0.77 (0.61–0.98)	.03	0.97 (0.85–1.13)	.74	0.87 (0.69–1.12)	.26
Alfa receptor blocker	1.13 (0.95–1.35)	.17	0.98 (0.72–1.34)	.89	1.04 (0.86–1.24)	.70	1.04 (0.75–1.42)	.83
Diuretic	1.27 (1.10–1.48)	.001	1.42 (1.10–1.83)	.006	1.18 (1.01–1.37)	.03	1.13 (0.87–1.46)	.36
Antiplatelet agent	1.25 (1.08–1.43)	.002	1.84 (1.46–2.33)	<.001	1.06 (0.91–1.23)	.47	1.29 (1.01–1.64)	.04
Warfarin or DOAC	1.01 (0.84–1.21)	.92	1.39 (1.05–1.83)	.02	1.06 (0.88–1.27)	.56	1.13 (0.85–1.50)	.40
Oral antidiabetic drugs	1.17 (0.97–1.41)	.12	1.35 (0.99–1.82)	.06	1.17 (0.95–1.43)	.14	1.27 (0.92–1.75)	.15
Insulin	1.51 (1.31–.75)	<.001	1.69 (1.33–2.15)	<.001	1.36 (1.12–1.64)	.002	1.41 (1.04–1.90)	.03
Antidepressant drug	1.22 (1.02–1.46)	.03	1.21 (0.90–1.63)	.21	1.10 (0.92–1.32)	.31	1.09 (0.80–1.475)	.58
Statin	1.06 (0.92–1.23)	.40	1.12 (0.88–1.42)	.36	1.03 (0.88–1.20)	.72	0.97 (0.75–1.24)	.80
PPI	1.55 (1.35–1.78)	<.001	1.85 (1.46–2.34)	<.001	1.32 (1.13–1.51)	<.001	1.47 (1.15–1.88)	.002

Clinical outcomes are due to COVID-19. P is considered significant at *P* ≤ .05.

^a^Multivariate analysis adjusted for age, sex, primary kidney disease, CCI score and CKD stage/dialysis.

ARB, angiotensin II-receptor blocker; DOAC, direct oral anticoagulants.

## DISCUSSION

In this nationwide cohort study of nephrology-referred patients with CKD stage 3b–5 or dialysis, 1.2%–3.6% died due to COVID-19 during the first 2 years of the pandemic. We found that patients with later stages of CKD and dialysis were at increased risk of COVID-19-related hospitalization and mortality compared with patients with CKD stage 3b. Primary kidney disease and comorbidity burden could not explain the association. In our CKD population, most comorbid conditions were associated with an increased risk of severe COVID-19, and overall comorbidity burden was one of the most prominent risk factors. CKD patients treated with insulin, PPI, diuretics, antiplatelets or immunosuppressants were at higher risk of severe COVID-19.

Previous epidemiological studies have consistently highlighted CKD as a major risk factor for COVID-19 severity [9, 17]. However, most studies have included CKD as a single entity, and few have considered CKD as a spectrum from mildly to severely reduced kidney function.

In our study, the risk of severe COVID-19 increased with worsening CKD stage. A similar relationship between degree of kidney impairment and COVID-19-related mortality was presented by Carter *et al*. [18]. In their study of older patients hospitalized for COVID-19, the eGFR at admission significantly correlated to the 28-day mortality. However, as the eGFR was registered at hospital admission, the study could not discriminate between an eGFR due to acute or chronic kidney injury. Two other studies have investigated the relationship between eGFR and COVID-19 prognosis based on preadmission creatinine values. Appelman *et al*. found higher ORs for 12-week mortality in patients with CKD stage 3 and 4 hospitalized for COVID-19 compared with patients without CKD, with ORs increasing with CKD stage [10]. Their results were not statistically significant for patients with CKD stage 5 or on dialysis, likely due to the small sizes of these cohorts. In a Danish study of hospitalized patients with COVID-19, a progressive 60-day risk of mortality with decreasing eGFR was observed in patients with preadmission eGFR 30–60 mL/min [11]. Both these studies were limited by the small number of included CKD patients. In our study we confirm and extend these findings by including a nationwide CKD cohort, demonstrating that the risk of severe COVID-19 increases with CKD stage also in patients with later stages of CKD, including dialysis.

The possible mechanisms underlying the progressive risk of severe COVID-19 with decreasing eGFR are likely to be multifactorial. As we did not limit our inclusion to COVID-19-positive cases, our results could reflect both an increased risk of acquiring COVID-19 and risk of developing severe disease when infected. Uremia has been associated with reduced lymphocyte production and impaired neutrophil functions, resulting in a generalized immunosuppressive state in end-stage CKD [19]. Since kidney disease is a continuum, it is likely that these alterations in immune cell function develop already in earlier stages. Furthermore, late-stage CKD necessitates closer monitoring at nephrology clinics, thus increasing the risk of COVID-19 exposure within hospital facilities as CKD advances. Due to shortage of hospital resources early in the pandemic, it is also possible that patients with late-stage CKD were not prioritized for critical care.

Although HD patients have been the focus of several studies during the pandemic, little light has been shed on PD patients. To date, the studies that have compared the rates of severe COVID-19 between the two dialysis modalities are few, small in scale, and lack adjustments for possible cofounders [20–22]. In our large cohort of dialysis patients, the higher ORs for hospitalization initially observed for HD patients attenuated slightly and lost significance after adjustments for confounders. However, mortality due to COVID-19 did not significantly differ between the two groups in either unadjusted or adjusted analyses.

Apart from CKD stage, a high CCI score ≥5 was the strongest risk factor for both hospitalization and death in our CKD population. Among individual comorbidities, chronic heart disease and diabetes, both of which are prevalent among CKD patients, as well as chronic pulmonary disease, liver disease and dementia, showed the strongest associations with COVID-19-related hospitalization. These findings are consistent with risk factors for severe COVID-19 in the general population [9]. Interestingly, presence of dementia strongly correlated with mortality, but was not significantly associated with hospitalization, possibly reflecting prioritization strategies applied early in the pandemic when hospital resources and beds were limited.

Aside from insulin, PPI was the only medication associated with increased risk of both hospitalization and mortality. In line with these results, a recent metanalysis reported a marginal, but statistically significant, increase in risk of severe COVID-19 associated with PPI use [23]. Whether PPI *per se* increases the risk of severe COVID-19, or if it serves as a surrogate marker for polypharmacy and multimorbidity, remains unanswered. Similarly, it is unclear whether insulin, diuretics and antiplatelets are true risk factors for severe COVID-19 in our population, or whether they are indicators of more severe underlying comorbid conditions. Early in the pandemic there was debate regarding the contribution of angiotensin-converting enzyme inhibitors (ACE-I) to COVID-19 mortality. In our study, use of ACE-I did not increase the risk of severe COVID-19, which is consistent with more recent evidence [24].

The main strengths of our study were the large sample size, the long follow-up period, and the nationwide approach. By linking multiple registries with high level of national coverage, we accessed detailed data on CKD, comorbidity, drug use and COVID-19-related outcomes. This minimized selection bias and allowed for adjustment for a wide range of confounders.

We also acknowledge some study limitations. First, the observational nature does not allow for conclusions regarding causality and leaves room for residual confounding. Although controlled for a variety of covariates, we could not obtain information on obesity, smoking and frailty—risk factors that could influence COVID-19 severity. Secondly, we only included patients registered in the SRR, hence leaving out CKD patients that have not been referred to specialist care. The included population may thus differ from the primary care CKD population in terms of age, CKD progression rate and comorbidity, in turn affecting the generalizability of our results. Third, categorizing patients into CKD stages and dialysis modalities at study start and excluding those without a registered creatinine value within 6 months of index date may have introduced misclassification and selection bias, as patients might have progressed in CKD, received a kidney transplant or switched dialysis modality during the observation time. However, we believe this misclassification bias to be small, and if anything, to underestimate the risk of severe COVID-19 in later CKD stages. The selection of patients with more recent creatinine values did not likely affect our results given the small number of excluded individuals, and the fact that some clinics in Sweden only registered out-patient visits once a year in all their patients.

In conclusion, we show that worsening CKD stage is an independent risk factor for COVID-19-related hospitalization and mortality, irrespective of comorbidity burden. Furthermore, overall comorbidity burden, as well as treatment with insulin, PPI, diuretics, antiplatelets and immunosuppressants, increases the risk of severe COVID-19 in patients with eGFR <45 mL/min. We call for enhanced awareness of severe COVID-19 in the CKD population. In the event of future surges in COVID-19, or the rise of other viral pandemics, our results could be used to guide and prioritize vaccination, novel therapies and healthcare resources to CKD patients at particular risk.

## Supplementary Material

sfad283_Supplemental_File

## Data Availability

The data underlying this article will be shared on reasonable request to the corresponding author.
