# Supplementary material for: Risk factors for COVID-19 hospitalization and mortality in patients with chronic kidney disease: a nationwide cohort study
Source: Clin Kidney J. 2023 Nov 28;17(1):sfad283. doi: 10.1093/ckj/sfad283 (PMC10768790; doi:10.1093/ckj/sfad283)
Supplement: sfad283_Supplemental_File [file sfad283_supplemental_file.docx]

**SUPPLEMENTARY MATERIAL**

**Table S1.** ICD-10 codes of included comorbidities.

| **Comorbid condition** | **ICD-10 code beginning with** |
| --- | --- |
| Coronary artery disease | I20-I25 |
| Congestive heart failure | I11, I50 |
| Peripheral vascular disease | I73, I74, I77 |
| Cerebrovascular disease | G45, G46, I60-I69 |
| Hemiplegia | G81-G82 |
| Dementia | F00-F03, G30 |
| Chronic pulmonary disease | J40-J47, J60-J66 |
| Diabetes without complications | E10-E14 (0.9) |
| Diabetes with end-organ damage | E10-E-14 (0.0-0.8) |
| Connective tissue disorder | M05-M08, M30-M36 |
| Mild liver disease | B18, K70, K73, K75 |
| Moderate to severe liver disease (cirrhosis) | K72, K74, I85 |
| Peptic ulcer disease | K25-K28 |
| Leukemia, lymphoma, multiple myeloma (<5 years) | C81-C96 |
| Solid tumour without metastasis | C00-C76 |
| Solid metastatic tumour | C77-C80 |
| HIV | B20-B24 |

*Abbreviations:* ICD, International Classification of Diseases.

**Table S2**. ATC-codes of included medications.

| **Medication** | **ATC code beginning with** |
| --- | --- |
| Systemic corticosteroid | H02 |
| Other immunosuppressive drugs | Tacrolimus (L04AD02), Cyclosporine (L04AD01), Mycophenolate mofetil (L04AA06), Azathioprine (L04AX01), Everolimus (L04AA18), Sirolimus (L04AA10), Methotrexate (L04AX03) |
| ACE-I or ARB | C09A, C09B, C09C, C09D, C09X |
| Beta blocker | C07A, C07B, C07C, C07D, C07E, C07F |
| Calcium channel blocker | C08C, C08D, C08E, C08G |
| Alfa blocker | C02C |
| Diuretic | C03A, C03B, C03C, C03D, C03E |
| Antiplatelet inhibitor | B01AC06 B01AC04 B01AC22, B01AC24, B01AC07 |
| Warfarin or DOAC | B01AA03 B01AF02, B01AE07, B01AF03, B01AF01 |
| Oral antidiabetic drug | A10B |
| Insulin | A10A |
| Antidepressant | N06A |
| Statin | C10AA |
| Proton pump inhibitor | A02BC |

*Abbreviations:* ATC, Anatomical Therapeutic Chemical codes; DOAC, direct oral anticoagulants; ACE-I, angiotensin-converting enzyme inhibitor; ARB, angiotensin II-receptor blocker.

**Table S3**. Median follow-up time until Covid-19 related hospitalization (or death before hospitalization) or Covid-19 related death.

|  | Median follow-up time (days) | |
| --- | --- | --- |
|  | Hospitalization | Death |
| CKD 3B | 704 | 716 |
| CKD 4 | 692 | 707 |
| CKD 5 | 666 | 683 |
| Dialysis | 583 | 610 |
| Total | 654 | 673 |

**Table S4**. Total number of deaths, deaths due to Covid-19 and deaths due to other causes.

|  | Total deaths *N* (%) | Covid-19 deaths *N* (%) | Non-Covid-19 deaths *N* (%) | Proportion of Covid-19 mortality in relation to overall mortality % |
| --- | --- | --- | --- | --- |
| CKD 3B | 290 (13.3) | 26 (1.2) | 264 (12.1) | 9.0 |
| CKD 4 | 814 (19.2) | 91 (2.2) | 723 (17.1) | 11.2 |
| CKD 5 | 404 (28.2) | 28 (2.0) | 376 (26.2) | 6.9 |
| Dialysis | 1201 (29.9) | 146 (3.6) | 1055 (26.3) | 12.2 |
